# Coagulation Changes during Presyncope and Recovery

**DOI:** 10.1371/journal.pone.0042221

**Published:** 2012-08-02

**Authors:** Gerhard Cvirn, Axel Schlagenhauf, Bettina Leschnik, Martin Koestenberger, Andreas Roessler, Andreas Jantscher, Karoline Vrecko, Guenther Juergens, Helmut Hinghofer-Szalkay, Nandu Goswami

**Affiliations:** 1 Institute of Physiological Chemistry, Medical University of Graz, Graz, Austria; 2 Department of Paediatrics, Medical University of Graz, Graz, Austria; 3 Institute of Physiology, Medical University of Graz, Graz, Austria; National Institutes of Health, United States of America

## Abstract

Orthostatic stress activates the coagulation system. The extent of coagulation activation with full orthostatic load leading to presyncope is unknown. We examined in 7 healthy males whether presyncope, using a combination of head up tilt (HUT) and lower body negative pressure (LBNP), leads to coagulation changes as well as in the return to baseline during recovery. Coagulation responses (whole blood thrombelastometry, whole blood platelet aggregation, endogenous thrombin potential, markers of endothelial activation and thrombin generation), blood cell counts and plasma mass density (for volume changes) were measured before, during, and 20 min after the orthostatic stress. Maximum orthostatic load led to a 25% plasma volume loss. Blood cell counts, prothrombin levels, thrombin peak, endogenous thrombin potential, and tissue factor pathway inhibitor levels increased during the protocol, commensurable with hemoconcentration. The markers of endothelial activation (tissue factor, tissue plasminogen activator), and thrombin generation (F1+2, prothrombin fragments 1 and 2, and TAT, thrombin-antithrombin complex) increased to an extent far beyond the hemoconcentration effect. During recovery, the markers of endothelial activation returned to initial supine values, but F1+2 and TAT remained elevated, suggestive of increased coagulability. Our findings of increased coagulability at 20 min of recovery from presyncope may have greater clinical significance than short-term procoagulant changes observed during standing. While our experiments were conducted in healthy subjects, the observed hypercoagulability during graded orthostatic challenge, at presyncope and in recovery may be an important risk factor particularly for patients already at high risk for thromboembolic events (e.g. those with coronary heart disease, atherosclerosis or hypertensives).

## Introduction

Recently, orthostatic stress has been shown to cause activation of the coagulation system [1]. In an upright posture, hematocrit in blood from the lower limbs is higher compared with that collected from the upper limbs [2], because increased filtration pressure causes transfer of intravascular fluid into surrounding tissues. This leads to systemic hemoconcentration with increased plasma protein concentration, hematocrit, and blood viscosity [3]–[6]. The increase in viscosity could contribute to activation of the coagulation system through various mechanisms, for example increased shear stress [3], [7]. Under unstressed conditions, endothelial cells have balanced anticoagulant and procoagulant properties [8]. Evidence suggests that a net elevation of local intravascular pressures and shear stress, as caused by prolonged standing, tips the balance towards procoagulant activity [5], [9].

Up to now, studies investigating the effects of orthostatic stress on the coagulation system have examined active standing. It was, therefore, the aim of our study to examine whether graded orthostatic stress affects coagulation changes as well as how rapidly coagulation parameters return to baseline values during recovery. A combination of head-up tilt (HUT) and lower body negative pressure (LBNP) was used to achieve presyncope. Seven male subjects were enrolled in our study.

All previous studies dealing with orthostatic stress and coagulation activation were carried out in platelet poor plasma (PPP) samples. However, while PPP contains a majority of the coagulation factors implicated in the coagulation process, whole blood includes phospholipid bearing cells and platelets with an important ability to support coagulation. Therefore, in the present study, thrombelastometry and platelet aggregation measurements were carried out in whole blood samples. For further hemostatic profiling assessment, prothrombin levels, endogenous thrombin potential, markers of endothelial activation (tissue factor, *TF*; tissue factor pathway inhibitor, *TFPI*, tissue-plasminogen activator, *t-PA*) and markers of thrombin generation (prothrombin fragment 1+2, F 1+2; thrombin/antithrombin-complex, *TAT*) were determined in PPP samples.

## Materials and Methods

### Subjects

Seven male subjects not using drugs known to affect the coagulation system were enrolled in this study. They were non-smokers, with no personal or family history of hypercoagulability, cancer, thrombosis, syncope or orthostatic intolerance.

### Experimental Design

All investigational procedures were performed in a human physiological laboratory under controlled environmental conditions in a quiet and partially darkened room with an ambient temperature of 24°C between 9 and 11 a.m. Heart rate and blood pressure were monitored continuously by using a TFM system (Task Force Monitor®, CNSystems, Graz, Austria). Each test started with a 30-min supine rest period to acquire cardiovascular data in steady-state conditions [10]. Tests were carried out using a tilt-table-LBNP device equipped with an adjustable footrest. LBNP sealing was positioned at the iliac crest [11]. To avoid erroneous readings due to hydrostatic effects during head up tilt, the lower arm was positioned at the heart level [12].

Monitoring of blood pressure and heart rate (3-lead ECG) was commenced during the supine control phase using both intermittent brachial cuff and continuous finger measurements (Task Force® Monitor, CNSystems, Graz, Austria). Subjects were instructed to avoid leg movements and to breathe normally. They were secured and had access to an emergency shutdown (automatic return to supine and pressure neutralization) at all times. The execution of the pre-programmed test protocol and synchronous data recording was performed using LabView® (National Instruments, Austin, TX, USA).

### Head-up Tilt and Graded LBNP Protocol

**Figure 1:**
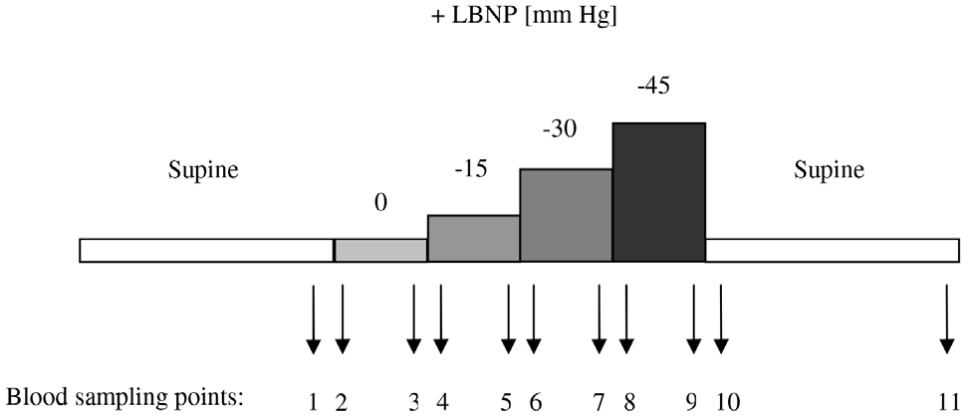
Test design and study protocol. Control blood was taken after 30 min supine rest (sample 1). The orthostatic stimulation phase started with passive 70° head-up for 4 min, then 15 mm Hg LBNP was commenced in this position and increased by 15 mm Hg every 4 min. After application of −45 mm Hg suction or after development of dizziness, LBNP was stopped and the table tilted back. Supine position was maintained for 20 min, at the end of which the last blood sample was taken (sampling point 11).

The study protocol and the blood sampling times are shown in **Figure 1**.

Each test started with the insertion of a 17G-1.4×40 mm Teflon® catheter followed by a 30 min supine rest period. At minute zero, the tilt table was brought from 0° (supine) to 70° head-up position for 4 min, after which pressure in the LBNP chamber was reduced to −15, −30, and −45 mm Hg every 4 min [13]. Each test ended with a 20 min post-LBNP supine rest period.

The local ethics committee approved the protocol and written informed consent was obtained.

### Blood Sampling

At each sampling point (**Figure 1**) two fractions of blood (each 4.5 mL) were collected from the uncongested vein into pre-citrated Vacuette® marked tubes (Greiner Bio-one GmbH, Kremsmünster, Austria), containing 500 µl of 3.8% sodium citrate. Another fraction (4 mL) was drawn into pre-heparinized syringes for high precision densitometry [4].

### Treatment of Blood Samples

Heparinized blood was immediately put on ice and spun for 15 min at 1500 g and 4°C (Ivan Sorvall, New York, NY, USA, RC2-B). The obtained plasma was used for plasma mass densitometry. Citrated blood samples were analyzed within 3 hours after collection (thrombelastometry, whole blood platelet aggregation, [14]). An aliquot was centrifuged at room temperature for 15 min at 1200 g to obtain PPP for subsequent determination of pro- and anticoagulant proteins, thrombin generation curves, and two markers of thrombin generation.

### Plasma Mass Density, Relative Changes in Plasma Volume and Hematocrit

Plasma mass density was measured with a DMA 602 MW (Paar KG, Graz, Austria). Relative changes in plasma volume were calculated as described previously [15]. Hematocrit was measured in duplicate by a standard technique.

### Whole Blood Thrombelastometry (TEM)

TEM was performed on a coagulation analyzer controlled by a portable computer. Using the thrombelastograph, we obtained the following values: Coagulation Time (CT), the period of time from initiation of the test to the initial fibrin formation; Clot Formation Time (CFT), time of beginning of clot formation until the amplitude of thrombelastogram reaches 20 mm; Maximum Clot Firmness (MCF), expressing the maximum strength in millimeters of the final clot; and Alpha, the angle between the line in the middle of the TEM tracing and the line tangential to the developing “body” of the TEM tracing. The alpha angle represents the acceleration (kinetics) of fibrin build up and cross-linking. Sample volume was 340 µl. Three hundred twenty µL of whole blood were re-calcified by addition of 20 µL of 0.2 M CaCl_2_. Under these experimental conditions clotting of whole blood is induced by the blood-born TF shed into the circulation from activated endothelial cells.

### Whole Blood (WB) Platelet Aggregation Assay

WB aggregation was assayed with WB aggregometer by the impedance method [16]. The increase in impedance upon stimulation is directly proportional to the platelet mass deposited on the electrode probe assembly. Impedance aggregometry results are expressed as “amplitude (or maximum aggregation) [ohm]” at 6 minutes after reagent addition and as “lag time (or aggregation time) [seconds]”, the time interval until the onset of platelet aggregation. The rate of platelet aggregation is expressed as “slope [ohm/min]”: a tangent is drawn through the steepest part of the aggregation curve. A right triangle is then constructed over an interval of one minute. The height of the triangle is defined as “slope”. Sample volume was 1 ml, 500 µl of citrated WB were incubated with 500 µl saline. Subsequently, platelet aggregation was provoked by addition of 2 µl collagen (2 µg/ml final concentration).

### Automated Fluorogenic Measurement of the Thrombin Generation

Measurement of the thrombin generation was performed using calibrated automated thrombography (CAT, [17]). For each experiment a fresh mixture of 2625 µl fluobuffer and 300 µl of 1 mol/l CaCl_2_ solution was prepared and incubated for 5 minutes at 37°C. After 5 minutes 75 µl of the Fluo-DMSO-solution were added, mixed and incubated for 5 minutes again. The resulting clear solution was referred to as FluCa. PPP-reagent as well as the thrombin calibrator was solubilized with 1 ml deionized water respectively. The thrombin calibrator was used in each experiment to compare the simultaneously measured thrombin activity in the sample to that from a known and stable concentration in the calibrator well. Either twenty µl of TF containing trigger solution (PPP-reagent) or thrombin calibrator were put into each sample well of a 96-well round-bottom microtiter plate made of polypropylene, purchased from Nunc, Roskilde, Denmark. Finally, 80 µl of PPP were put into each well. All reagents were warmed up to 37°C before starting the experiment. The 96-well-plate was placed in the fluorometer (Fluoroskan Ascent, Thermolabsystems OY, Helsinki, Finland) with an excitation filter at 390 nm and an emission filter at 460 nm. The automated dispensing of 20 µl FluCa started the measurement process. During 60 minutes each well was measured every 20 seconds. Each experiment was performed three times. Upon completion of the measurement we used the Analysis Software from Thrombinoscope BV to analyze our results. The ability of a given plasma sample to generate thrombin was assessed with respect to lag time preceding the thrombin burst (Lag Time), time to peak (ttPeak), peak height (Peak), and endogenous thrombin potential (ETP), and the time point at which free thrombin has disappeared (StartTail). Measurements were carried out in the presence of low amounts (5 pmol/l final concentration) of tissue factor (TF), a condition that allows sensitive detection of thrombin formation.

### Standard Laboratory Tests

Determination of prothrombin levels (F II) was performed on a BM/Hitachi 917 from Roche (Vienna, Austria). Tissue Factor, Tissue Factor Pathway Inhibitor, and tissue-Plasminogen Activator were determined by means of the assays ACTICHROME® Tissue Factor, ACTICHROME® TFPI Activity, and IMUBIND tPA ELISA kit, respectively, from American Diagnostica (Pfungstadt, Germany). Plasma levels of Prothrombin fragment 1+2 (F 1+2) and Thrombin-Antithrombin complexes (TAT) were determined by means of ELISA kits from Behring Diagnostics GmbH (Marburg, Germany).

### Catecholamines

Baseline and presyncopal levels of catecholamines were measured with HPLC using electrochemical detection after prior alumina extraction (Chromsystems, FRG, [18]).

### Reagents and Devices

Blood cell counts were determined on a Sysmex KX-21N Automated Hematology Analyzer from Sysmex (Illinois, USA). Fluobuffer for monitoring thrombin generation curves contained 20 mmol/l HEPES and 60-mg/ml bovine serum albumin, both purchased from Sigma, St. Louis, Mo., USA. Working buffer consisted of 140 mmol/l NaCl, purchased from Merck, Darmstadt, Germany, 20 mmol/l HEPES, and 5 mg/ml human serum albumin, purchased from Sigma, St. Louis, Mo., USA. The fluorogenic substrate Z-Gly-Gly-Arg-amino-methyl-coumarin was purchased from Bachem, Bubendorf, Switzerland, and was solubilized in pure dimethylsulfoxide (DMSO), which was purchased from Sigma, St. Louis, Mo., USA. Calcium chloride was purchased from Merck, Darmstadt, Germany. The platelet-poor plasma (PPP) reagent with a content of 5 pmol/l TF and 4 µmol/l phospholipids in the final reaction mixture, and the thrombin calibrator was purchased from Thrombinoscope BV, Maastricht, the Netherlands. The TEM coagulation analyser (ROTEM^®^05) was purchased from Matel Medizintechnik, Graz, Austria. Whole-blood aggregation experiments were performed on the Chrono-Log Whole Blood Aggregometer Model 590 from Probe & Go (Endingen, Germany). The Chrono-Par reagents collagen and saline (0.85%, w/v) were also purchased from Probe & Go.

### Statistics

Calculations were performed by SPSS 18.0 (SPSS Inc., Chicago, Illinois, USA). Data are presented as mean ± SD. A non-parametric paired Wilcoxon test was used to compare the obtained coagulation values at supine rest, presyncope and 20-min post-stress [19]. A P-value less than 0.05 was considered as statistically significant.

## Results

The study criteria were met by 7 male participants of age 26.8±1.4 years, weight 72±7.4 kg, height 180.1±3.8 cm (mean ± SD).

Using the HUT + graded LBNP, presyncope was achieved in all the subjects. Four participants developed dizziness at −45 mm Hg, three at −30 mm Hg, and one at −15 mm Hg suction. They were brought immediately to supine rest.

### Plasma Shift

**Figure 2:**
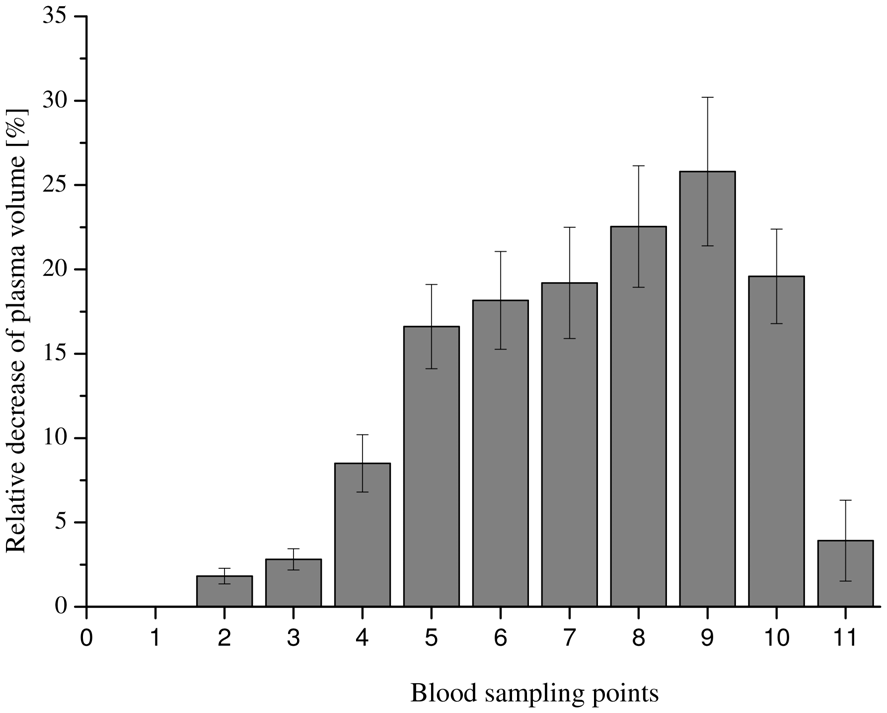
Relative change in plasma volume. The effect of HUT/LBNP on hemoconcentration is shown with respect to the relative change of the plasma volume. Data are presented as mean ± SD.

Relative changes in plasma volume were calculated from changes in plasma density, according to our previous work [15]. In all participants, plasma volume decreased with increasing suction, shown in Figure 2. Plasma volume was approximately 25% below the initial supine value at −45 mm Hg suction. At the end of the post-LBNP recovery period, plasma volumes reached approximately the pre-LBNP levels in all participants.

### Effects of HUT/LBNP on Blood Cell Counts

At the end of the initial 20 min supine rest period, participants’ WBC count was 5.05±1.25 · 10^3^/ µL, RBC count was 4.57±0.39 · 10^6^/ µL, and platelet count was 216±40·10^3^/ µL. In all participants, WBC, RBC as well as platelet counts increased during head-up/LBNP, at an extent completely commensurable with hemoconcentration. Baseline (supine) levels were reached at the end of the recovery period in all participants.

### Effects of HUT/LBNP on Thrombelastometry Values

The HUT/LBNP procedure did not cause significant alterations of the thrombelastometry values. Interestingly, a procedure-related shortening of CTs (P = 0.087) and a procedure-related increase of MCF (P = 0.055) at maximum suction compared to initial supine rest showed a tendency towards significance (**Table 1**).

**Table 1:** Effects of HUT/LBNP on coagulation values.

	Initial supine rest	HUT/LBNP (−45 mm Hg)	End of post-procedure recovery period
Thrombelastometry:			
CT [s]	627.3±171.4	621.8±180.5	548.5±218.4
CFT [s]	303.5±95.3	323.4±116.5	326.9±115.3
MCF [mm]	44.3±5.4	48.5±3.4	45.1±3.4
Alpha [°]	44.2±10.7	41.2±11.2	44.4±11.7
Platelet aggregation:			
Lag time [s]	100.6±16.3	132.3±22.6*	165.5±38.7**
Slope [Ohm/min]	5.8±1.2	6.8±1.0*	5.86±0.9
Amplitude [Ohm]	11.5±2.3	11.3±1.2	10.0±1.7
Thrombin generation:			
Peak [nmol/L]	359.7±54.3	402.9±63.3***	362.5±57.7
ETP [nmol/L · min]	1755.9±239.3	1863.9±264.8*	1693.4±203.2
Standard coagulation values:			
F II [%]	102.5±11.2	116.8±11.7****	106.0±13.9
TFPI [ng/mL]	40.0±14.7	52.4±9.6*	45.5±9.7
t-PA [ng/mL]	3.2±1.9	5.15±1.9***	3.8±2.3
TF [pg/mL]	357.4±158.4	555.3±307.1*	338.6±136.3
F 1+2 [mmol/L]	385.7±168.4	590.7±373.5*	1007.2±546.5**
TAT [ng/mL]	31.7±16.7	45.8±22.9**	88.1±37.8**

A non-parametric paired Wilcoxon test was used to compare the obtained coagulation values at supine rest, presyncope, and 20-min post-stress (20).

* …P<0.05;

** …P<0.01;

*** …P<0.001,

**** …P<0.0001,

compared to initial supine rest. Data are presented as mean ± SD.

### Effects of HUT/LBNP on WB Platelet Aggregation Values

The head-up tilt/LBNP procedure caused a significant prolongation of “Lag Times” at maximum suction (P = 0.024) and also at the end of the post-LBNP recovery period (P = 0.0081) compared to the initial supine rest baseline data. “Slope” was significantly increased at maximum suction (P = 0.042) and returned to baseline levels at the end of the post-LBNP recovery period. A procedure-related decrease of “Amplitude” at the end of the post-LBNP recovery period showed a tendency towards significance (P = 0.051, **Table 1**).

### Effects of HUT/LBNP on Thrombin Generation Parameters Evaluated by Means of CAT

**Figure 3:**
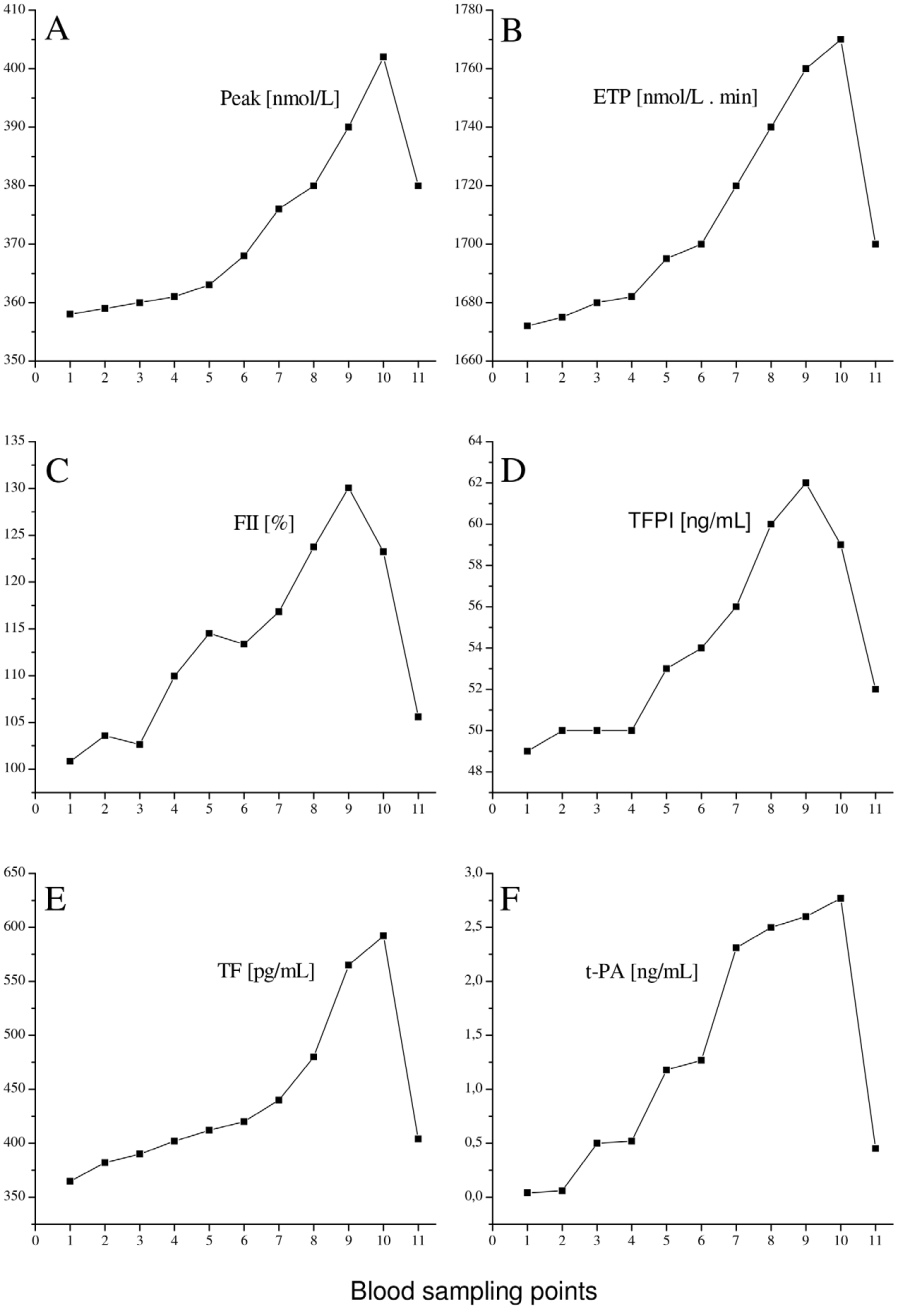
Effect of HUT/LBNP on coagulation values. The effect of HUT/LBNP on thrombin Peak (*panel A*) and on ETP (*panel B*), both evaluated by means of CAT, and on plasma levels of prothrombin (*panel C*) and TFPI (*panel D*), as well as on TF (*panel E*) and on t-PA (*panel F*) are shown. Each graph represents a representative experiment.

At the end of the initial 30 min supine rest period, Lag time was 2.13±0.32 min, ttPeak was 4.15±0.57 min, and StartTail was 18.51±2.25 min and remained unchanged throughout the whole procedure. Peak and ETP significantly increased during the procedure and reached initial supine rest values at the end of the post-LBNP recovery period (**Table 1**). Two representative experiments are shown in **Figure 3**, panels A and B, respectively. The increase of Peak and ETP is completely commensurable with hemoconcentration.

### Effects of HUT/LBNP on Standard Laboratory Tests

Prothrombin and TFPI levels significantly increased during the procedure commensurable with hemoconcentration and returned to baseline levels at the end of the post-LBNP recovery period (**Table 1**). Two representative experiments are shown in **Figure 3**, panels C and D, respectively.

Plasma levels of TF and t-PA also significantly increased during the procedure but to an extent beyond the hemoconcentration effect. Both levels reached baseline values at the end of the post-LBNP recovery period (**Table 1**). Two representative experiments are shown in **Figure 3**, panels E and F, respectively.

**Figure 4:**
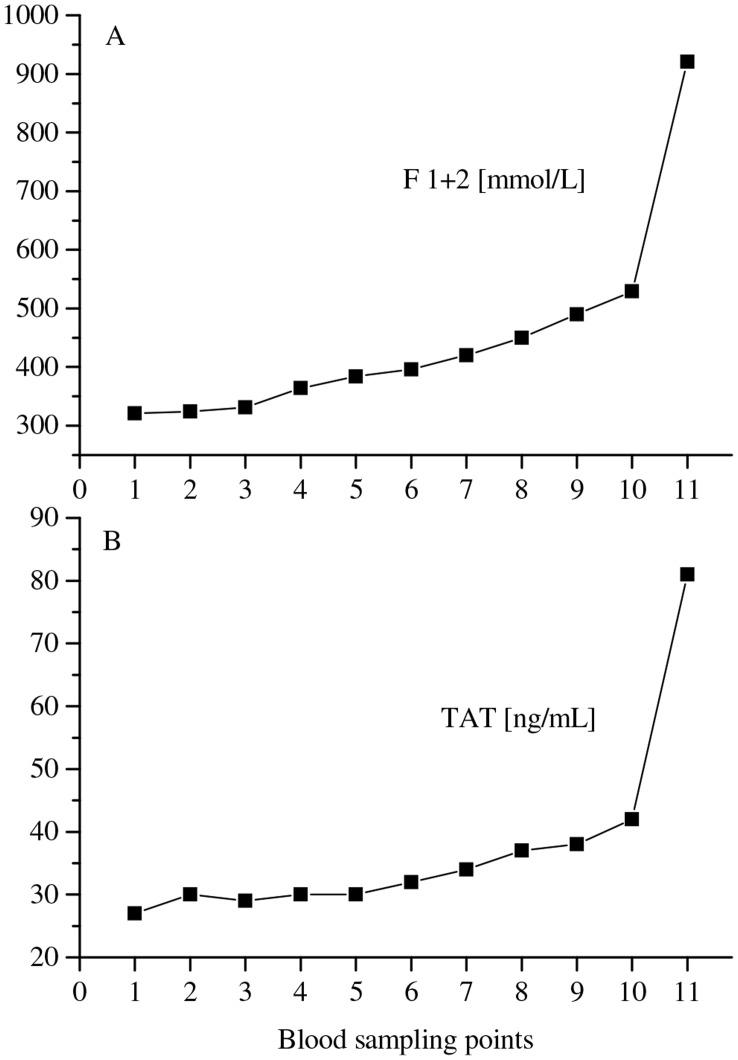
Effect of HUT/LBNP on thrombin generation values. The effect of HUT/LBNP on plasma levels of F1+2 (*panel A*) and on TAT (*panel B*) are shown. Each graph represents a representative experiment.

The markers of thrombin generation, F1+2 and TAT, also increased significantly during the procedure at an extent beyond the hemoconcentration effect, and, interestingly, did not decrease to baseline values but reached highest values at the end of the post-LBNP recovery period (**Table 1**). Two representative experiments are shown in **Figure 4**, panels A and B, respectively.

### Effects of HUT/LBNP on Catecholamine Levels

Plasma levels of norepinephrine, epinephrine and dopamine significantly increased during the procedure (**Table 2**), to an extent far beyond the hemoconcentration effect.

**Table 2:** Effects of HUT/LBNP on catecholamine levels.

	Initial supine rest	Presyncope
Norepinephrine [pg/mL]	193.9±22.4	1310.2±96.3****
Epinephrine [pg/mL]	28.2±4.3	80.8±9.1****
Dopamine [pg/mL]	15.9±3.2	29.1±6.2***

* …P<0.05;

*** …P<0.001,

**** …P<0.0001,

compared to initial supine rest. Data are presented as mean ± SD.

## Discussion

In the present study we investigated the effects of graded orthostatic stress on the coagulation system in 7 healthy male subjects. We found that plasma volume decreases significantly with increasing orthostatic exposure. For example, when HUT/LBNP (−45 mm Hg) was applied, plasma volume loss was approximately 25%. Comparatively, the relative mild stimulus of active standing has been shown to cause a plasma concentration of approximately 12% [1]. Commensurable with hemoconcentration, we found increasing levels of blood cells and of plasma prothrombin levels, TFPI, and CAT values (thrombin) Peak and ETP. All these values reached baseline levels at the end of the post-LBNP recovery period.

Furthermore, hemoconcentration has been shown to cause not only an increase in levels of plasma proteins but also an increase in blood viscosity, leading to increased shear stress acting on the endothelial monolayer of the vessel walls [6], [19] associated with activation of both the endothelium as well as of the coagulation cascade [9], [20]. Presumably, this should be true particularly under the extreme orthostatic load applied in our experiments (HUT/LBNP). Our results also suggest this. We observed a significant increase in the endothelial activation-related factors t-PA (up to 60%) and TF (up to 55%), an extent far beyond the hemoconcentration effect. In addition, the t-PA and TF levels decline to baseline values in the absence of orthostatic stress, i.e. at the end of the post-LBNP recovery period.

Moreover, the markers of thrombin formation were also significantly raised during the procedure far beyond the hemoconcentration effect. At HUT/LBNP (−45 mm Hg), F1+2 levels were increased by approximately 53%, and TAT levels by approximately 45%. Notably, the CAT-derived thrombin generation parameters Peak and ETP were only increased at an extent commensurable with hemoconcentration and reached baseline (supine) values at the end of the post-LBNP recovery period.

Interestingly, levels of F1+2 and TAT continued to increase even after the subjects were brought into supine position again (a state of no shears stress). We found markedly elevated levels of F1+2 (≅161%) and TAT (≅178%) compared to baseline values at the end of the post-LBNP recovery period. Since shear stress is terminated with the stop of challenge, other triggers have to be in place causing coagulation activation/thrombin generation during the post-LBNP recovery period. We speculate the following:

- Orthostatic stress and the period following termination of the orthostatic stress have been both shown to be associated with hormonal stimulation [18]–[19]. Could the elevated levels of hormones be responsible for the heightened coagulatory state? Using a similar protocol of presyncope, induced by HUT + graded LBNP, we have previously reported that 20 min post-presyncope, the levels of adrenocorticotropic hormone (ACTH) and Arginine vasopressin (AVP) are still elevated [19]. As ACTH is involved in the production of cortisol, and hypercortisolemia is associated with a hypercoagulable state [21], its role in the observed heightened coagulatory state post stress cannot be ruled out. Similarly, vasopressin levels are increased several fold at presyncope and stay elevated for long periods during recovery [19]. As vasopressin promotes platelet aggregation and agglutination [22], perhaps the heightened coagulatory state could be attributed to the high levels of vasopressin? The role of catecholamines also needs to be explored further. Even though post stress catecholamines levels return to baseline levels rather fast [23], the initial high levels of catecholamines 2–3 min post stress are still high [23]. Could they play important roles in the elevated F1+2 levels (**Figure 4**), at least in the initial stage of recovery from presyncope? Previous reports have shown that increased catecholamines are associated with increased coagulation activity [24]. In the future, the time course of recovery of these hormones should be studied more comprehensively using increased frequency of blood collections.
- The stress response has been reported to be non-specific [25] and leads to net hypercoagulability. The latter is believed to be of survival benefit from the evolutionary point of view, as it limited blood loss in our forefathers during the fight or flight response (discussed in [26]). Perhaps the observation of heightened coagulation, following central hypovolemia induced by graded HUT and LBNP, during recovery is simply a part of the generalized stress response.

Whatever the reason for heightened coagulatory state observed in our study, it is important to remember that hypercoagulable states might predispose susceptible subjects to thrombotic events. While our experiments were carried out in healthy controls, and as such did not trigger thrombotic events or myocardial ischemia, the hypercoagulability during recovery may predispose those with histories of hypertension [27], vascular diseases, and thrombotic events to thrombo-vascular events.

It therefore appears that not only the graded orthostatic stress but also the recovery period is equally important. For instance, Thrall et al. (2007) did a systematic review of the literature for the pathogenesis of acute coronary syndrome and concluded “individuals may be at a greater risk of stress induced thrombogenesis in the period *following* physical activity” [28]. Our findings of an increased coagulatory state at 20 min of recovery from presyncope may have greater clinical significance than short term procoagulant changes observed during standing [1]. We are not aware of any study that has studied prolonged recovery times following presyncope. Our results are also in agreement with the recent observations that recovery periods from stressors might be more relevant to the development of diseases than the actual stress itself (for details see [29]–[30]).

### Limitations

We found that orthostatic exposure causes prolonged lag times until the onset of platelet aggregation during and after the procedure indicating impaired platelet function. This is contradictory to the assumption that orthostatic stress leads a catecholamine-induced activation of platelets. A possible explanation might be that under the orthostatic stress-induced high shear stress platelets spontaneously activate, secret their products, form aggregates, de-aggregate, and then recirculate as exhausted defective platelets, similar to the findings of Michiels et al. in patients with thrombocythemia and polycythemia vera [31]. Therefore, the “activation status” of platelet prior, during, and after orthostatic expose should be examined in the future.

Another limitation of our study is that we have no data of the hormone profiles during 20 min post stress. However, previous studies done in our laboratory, using graded HUT + LBNP, have shown that the levels of vasopressin, ACTH are elevated 20 min post stress ([19], see above).

In conclusion, we studied the changes in the coagulation system in humans exposed to hypergravitational stress. Indeed the findings of our study emphasize the importance of the novel physiological mechanism “orthostatic procoagulation” [1] and that, more importantly, the pro-coagulant effect persists for up to 20 min after presyncope, which is the end point of cardiovascular stress. Even though this study was carried out in healthy controls, it could also serve as a warning to patients with high risk factor of thromboembolic events (e.g. those with coronary heart disease, atherosclerosis or hypertensive) to be not exposed to hypergravitational stress. Orthostatic stress has to be considered as important risk factor particularly in these patients since coagulation activation occurs not only during but also beyond the termination of the orthostatic challenge.

### Future Directions

The markers of thrombin generation (F1+2 and TAT) remained most highly elevated at the end of blood sampling points at 20 min post-LBNP supine rest. As the half-lives F1+2 and TAT are 90 min and 15 min, respectively [32], and our primary goal was not the post-loading time course, we speculate that longer periods of recovery should be assessed in the future.
